# The advanced lung cancer inflammation index predicts chemotherapy response and infection risk in multiple myeloma patients receiving induction chemotherapy

**DOI:** 10.3389/fgene.2022.1047326

**Published:** 2022-11-08

**Authors:** Jie Cheng, Qianyuan Li, Sheng Xiao, Lu Nie, Jianping Liao, Qingjie Jiang, Biyu Xiang, Hongfei Zhang, Yanhong Jiang, Chenjiao Yao

**Affiliations:** ^1^ The Third Xiangya Hospital of Central South University, Changsha, China; ^2^ The First Affiliated Hospital of Hainan Medical University, Haikou, China

**Keywords:** advanced lung cancer inflammation index, multiple myeloma, chemotherapy response, infection, overall survival (OS)

## Abstract

**Objective:** This study aims to determine the clinical significance of the advanced lung cancer inflammation index (ALI) in predicting prognosis, chemotherapy response, and infection risk in newly diagnosed multiple myeloma (MM) patients receiving induction therapy.

**Methods:** A retrospective analysis of the clinical characteristics and laboratory data of 111 newly diagnosed MM patients from the Haematology Department of the Third Xiangya Hospital of Central South University from January 2014 to March 2020 was performed. We first determined the relationship between ALI and overall survival (OS), as well as clinical and laboratory parameters. Second, predictive factors for chemotherapy response were analysed by univariate and multivariate regression analyses. Third, univariate regression analysis of risk factors was performed using infection as the evaluable outcome.

**Results:** Of the 111 evaluable patients, the low ALI group (<32.7) exhibited significantly poorer survival than the high ALI group (51 months versus 77 months). Multivariable analysis showed that advanced age, chemotherapy response and serum calcium level were independent prognostic factors for OS. Better chemotherapy efficacy in the high ALI group (89.3%) than in the low ALI group (42.2%) (*p* < 0.001) was noted. Multivariate analysis suggested that only ALI [HR: 0.110, 95% CI (0.035–0.350), *p* = 0.000] is an independent predictive factor in evaluating the efficiency of induction chemotherapy. Forty patients (36.04%) presented with infection after induction chemotherapy. Univariate analysis suggested that low ALI and abnormal renal function increase risk of infection in newly diagnosed MM patients.

**Conclusion:** Our study confirmed that ALI is not only a prognostic biomarker for newly diagnosed patients, but also predicts chemotherapy efficacy in newly diagnosed MM patients receiving induction therapy.

## Introduction

Multiple myeloma (MM) is the second most common malignant tumour of the haematological system, accounting for 1% of haematological tumours (Raab et al., 2009; Palumbo and Anderson, 2011). Revised International Staging System (R-ISS) and the Durie-Salmon (D&S) staging system have been established to predict survival based on prognostic classification of MM patients (Boyd et al., 2012; Palumbo et al., 2015). Induction therapy is the most crucial treatment in newly diagnosed multiple myeloma (NDMM) patients, and bortezomib (PI) and dexamethasone (VD) remain the current standard of care. The recently updated 2021 EHA-ESMO clinical practice guidelines recommend the use of either lenalidomide-VD (VRD) or daratumumab-thalidomide-VD (Dara-VTD) as first-line options for transplant-eligible NDMM patients; when not available, thalidomide-VD (VTD) or cyclophosphamide-VD (VCD) are acceptable alternatives (Bazarbachi et al., 2022). The majority of MM patients are in a refractory or relapsed state (Röllig et al., 2015). Clinical evidence confirms that infections represent a major cause of morbidity and mortality in patients with MM, especially for those with severe infection, pneumonia, and neutropenia in relapsed/refractory settings (Blimark et al., 2015; Balmaceda et al., 2021). In NDMM patients admitted for the first time, risk of infection correlates with poor prognosis, particularly in those with ISS stage III or low haemoglobin levels (Lin et al., 2020). MM patients with a risk of infection and complications are recommended to receive optimal preventive strategies (Bladé and Rosiñol, 2005; Raje et al., 2022). Additionally, biological mechanism research has demonstrated that the proinflammatory cytokine interleukin (IL)-18 is involved in tumour-promoting inflammation, and its high expression suggessts poor overall survival in MM (Nakamura et al., 2018). All of these results indicate that inflammatory markers have potential as prognostic markers (Hanahan and Weinberg, 2011). The inflammation-based neutrophil-to-lymphocyte ratio (NLR) has been demonstrated to be a prognostic biomarker, with higher NLR indicating poorer prognosis in MM patients, as demonstrated by meta-analysis (Mu et al., 2018). Similarly, the advanced lung cancer inflammation index (ALI), which is a modification of NLR that includes albumin (ALB) and body mass index (BMI), has been developed and demonstrated to be a prognostic marker of poor survival in several cancers, including non-small cell lung cancer (NLSCL) (Jafri et al., 2013). ALI also represents a simple tool to predict immunotherapy efficacy in patients with advanced NSCLC treated with PD-L1 inhibitors alone, and the ALI score has a stronger predictive effect than NLR, the PD-L1 tumour proportion score and other biomarkers (Shiroyama et al., 2018; Mountzios et al., 2021). Therefore, ALI, which is calculated based on body composition, nutrition, and systemic inflammation, may represent a comprehensive indicator for predicting prognosis of NDMM patients. Although ALI reflects the systemic inflammation and cachexia provoked by cancer, its prognostic and predictive value in NDMM patients is unknown. We hypothesized that low ALI is associated with poor survival and can predict chemotherapy efficacy in NDMM patients. We also sought to evaluate the predictive capacity of ALI on the overall incidence of infection in patients receiving induction chemotherapy.

## Materials and methods

### Patients and methods

This was a single-centre, observational, retrospective study. Our study collected data for 111 MM patients at the Department of Haematology, Third Xiangya Hospital, Central South University from January 2014 to March 2020 under approval of the Institutional Review Board of Third Xiangya Hospital, Central South University (No. 22081). We then collected clinical data and performed classification according to ISS staging standards and the DS staging system. Abnormal secretion of clonal immunoglobulins was classified into the following categories: IgG, IgD, IgM, IgE, double clonal, kappa, and lambda light chain or nonsecreting types. NDMM patients were treated consecutively with bortezomib-based triplet regimens, consisting of proteasome inhibitor (PI) bortezomib plus dexamethasone (VD) backbone, with the addition of a third agent, such as thalidomide (VTD), cyclophosphamide (VCD), lenalidomide (VRD) or doxorubicin (PAD). The number of induction cycles varied from 4 to 6. The curative effect was evaluated according to the traditional efficacy standard of International Myeloma Working Group in 2016 (Kumar et al., 2016). Complete remission in a strict sense (SCR), complete remission (CR), very good partial remission (VGPR), and partial remission (PR) are regarded as effectively curative effects; stable disease (SD) and progressive disease (PD) are regarded as ineffective curative effects. In addition, patients who meet any of the following conditions after initial diagnosis of chemotherapy should be judged as having complicated infection: 1) continuous fever for more than 2 days (axillary temperature greater than 38°C), and fever caused by blood transfusion or related drugs should be excluded; 2) exact clinical symptoms and/or signs of infection, such as bladder irritation, frequent micturition and urgent micturition or signs, such as tenderness of the ureter point when urinary system infection occurs; and 3) exact imaging-related examinations and/or aetiological evidence suggesting the existence of infected foci. The exclusion criteria were as follows: 1) acute or chronic inflammation at the time of initial diagnosis; 2) a history of blood system diseases and malignant tumours; 3) immune disease, such as inflammatory bowel disease, Sjogren’s syndrome, or rheumatoid arthritis; 4) complication of acute and chronic hepatitis or liver disease; 5) complication of severe damage to the functions of other important organs except the kidneys; 6) indicators, such as height and weight, not recorded at the time of admission; 7) relevant biochemical tests not completed within 48 h after admission; and 8) treatment discontinuation or incomplete clinical data.

Data were collected retrospectively from the electronic medical records. Weight, height, neutrophil, lymphocyte, and ALB (g/dl) data were collected at baseline before chemotherapy administration. BMI was calculated by dividing weight (kg) by height (m) squared. The neutrophil-to-lymphocyte ratio (NLR) was computed as the absolute neutrophil count divided by the absolute lymphocyte count. We calculated the advanced lung cancer inflammation index (ALI) as follows: ALI = BMI ×ALB/NLR.

### Statistical methods

The χ2 test or Fisher’s exact test was used to determine the significance of differences between discrete variables. Significant variables in univariate analysis were included in binary multivariate logistic analysis. A receiver operating characteristic curve (ROC) was used to determine specificity and sensitivity. Univariate and multivariate analyses were performed using the Cox proportional risk regression model to evaluate prognostic factors and their impact on OS. Associations between ALI and OS were analysed using Kaplan‒Meier survival curve estimates and compared using the log-rank test. The statistical analysis was performed using SPSS version 26.0, and *p* < 0.05 was considered statistically significant.

## Results

### Patient characteristics

Overall, we analysed 111 patients with NDMM at first hospitalization (66 males and 45 females). The average age was 58 years (44–75), and 39.6% of the patients were ≥60 years. A total of 49 patients (44.1%) had MM of the IgG type, 27% had the IgA type, and 18% had the *λ* light chain type. In total, 10.8% of the patients were classified with other types (κ light chain type and IgD type or others). Based on the Durie-Salmon scale, four patients were at stage I, 12 at stage II, and 95 at stage III. In total, 90 patients had normal renal function, whereas 21 had abnormal renal function. Based on the International Staging System (ISS) scale, 12 patients were at stage I, 28 at stage II, and 71 at stage III. All patients received two cycles of induction chemotherapy, and curative effects were evaluated. In total, the response in 78 patients was considered effective, whereas the response was classified as ineffective in 33 patients.

### Overall survival

**FIGURE 1:**
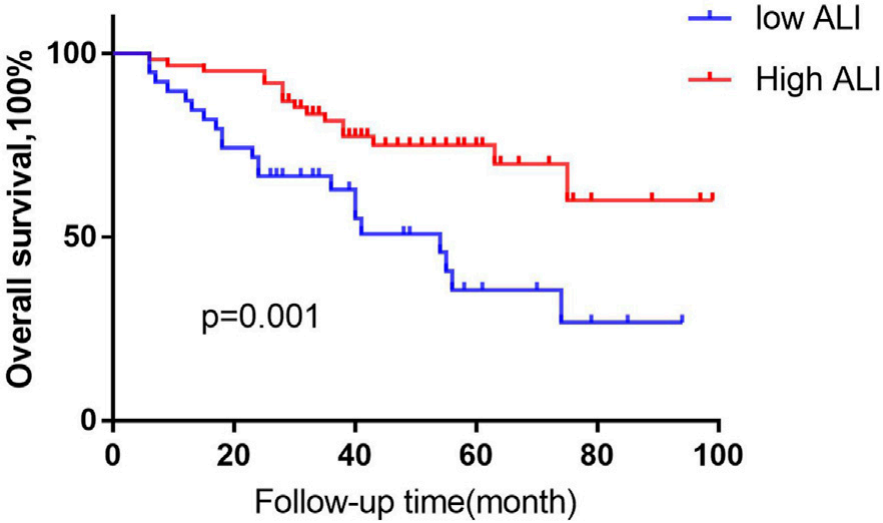
Kaplan-Meier analysis of OS based on ALI status. Using the X-tile program, an optimal cut-off value for ALI of 32.7 was determined. We defined MM patients the high- ALI group, (*n* = 66 cases) and the low -ALI group, (*n* = 45). Low-ALI patients with MM were significantly correlated with poor prognosis compared with high-ALI patients with respect to overall survival (OS; *p* = 0.001, log-rank test).

We first analysed the association between ALI and patient prognosis. Using the X-tile program (Camp et al., 2004), an optimal cut-off value for ALI of 32.7 was determined. All cases were categorized into the following two groups: a high ALI group (*n* = 66 cases) and a low ALI group (*n* = 45). Patients in the low ALI group had significantly poorer survival than those in the high ALI group (51 months versus 77 months, *p* = 0.001, Figure 1). Univariate analyses showed that OS correlated with NLR, abnormal renal function, advanced age (>60 years), *ß* 2-MG level, ISS stage, chemotherapy efficacy, and serum calcium level. Multivariable analyses showed that advanced age, chemotherapy response, and serum calcium were independent prognostic factors for OS (Table 1).

**TABLE 1:** Cox regression analysis of predictive factors for overall survival in patients with MM (*n* = 111).

Clinical characteristic	Univariate		Multivariate	
	HR (95% CI)	*p*-value	HR (95% CI)	*p*-value
Age (years)	0.440 (0.220, 0.843)	0.013*	0.300 (0.143, 0.631)	0.002*
Gender	0.686 (0.359, 1.311)	0.254		
Classification of disease	0.750 (0.381, 1.474)	0.403		
ISS stage	0.303 (0.107, 0.857)	0.024*	0.308 (0.300, 3.171)	0.322
DS stage	0.712 (0.217, 2.332)	0.574		
Renal function	2.167 (1.102, 4.263)	0.025*	0.675 (0.310, 1.472)	0.323
Plasma cell ratio	0.857 (0.453, 1.690)	0.692		
β2-MG(mg/L)	0.305 (0.138, 0.671)	0.003*	0.565 (0.158, 2.023)	0.381
Hb(g/L)	2.38 (0.995, 5.728)	0.051		
Chemotherapy response	2.05 (1.067, 3.951)	0.031*	2.199 (1.010, 4.788)	0.047*
ALB	1.062 (0.557, 2.025)	0.855		
Chemotherapy regimens	1.96 (0.972, 3.962)	0.060		
NLR	2.006 (1.030, 3.905)	0.041*	0.596 (0.247, 1.435)	0.248
ALI	0.362 (0.189, 0.696)	0.002*	1.192 (0.484, 2.937)	0.703
PLT	0.988 (0.502, 1.944)	0.972		
Ca	2.055 (1.010, 4.181)	0.047*	0.748 (0.337, 1.661)	0.047*

*p* < 0.05.

### Characteristics of laboratory results and the response to chemotherapy

**FIGURE 2:**
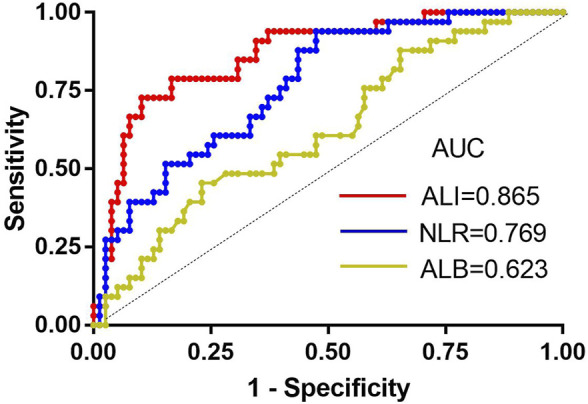
The receiver-operating characteristic (ROC) curves for ALI, ALB, and NLR. AUC, area under the curve; ALI, advanced lung cancer inflammation index; NLR, neutrophil-to-lymphocyte ratio; ALB, albumin.

Next, we analysed the relationship between ALI and patient characteristics as well as laboratory parameters. Table 2 shows that no differences in age, sex, disease classification, plasma cell ratio, ISS stage, DS stage, haemoglobin level, or *ß* 2-MG level were noted between the two groups. However, abnormal renal function and increased serum calcium were significantly associated with the low ALI group. Better chemotherapy efficacy was observed in the high ALI group (89.3%) than in the low ALI group (42.2%) (*p* < 0.001), but there was no significant difference between the high ALI group and the low ALI group in whether bortezomib was used (*p* > 0.05). In univariate analyses, abnormal renal function, lower ALI, and high NLR level was related to poor chemotherapy efficiency, but multivariate analysis suggested that only ALI [HR: 0.110, 95% CI (0.035–0.350), *p* = 0.000] was an independent predictive factor in evaluating the efficiency of induction chemotherapy (Table 3). We compared the predictive value of NLR, ALB, and ALI in patients with MM treated with chemotherapy. The areas under the ROC curves of NLR, ALB, and ALI were calculated (Figure 2), and ALI and NLR were significantly associated with OS. Notably, based on ROC curve analyses, ALI was identified as a more powerful prognostic marker than NLR and ALB.

**TABLE 2:** Baseline clinical characteristics according to the ALI.

Clinical characteristic	Total	ALI group		χ^2^	*p*
		High (*n* = 66)	Low (*n* = 45)		
Age (years)				0.004	0.949
<60	67	40 (60.6)	27 (0.0)		
≥60	44	26 (39.4)	18 (40.0)		
Gender				0.240	0.624
Male	66	38 (57.6)	28 (62.2)		
Female	45	28 (42.4)	17 (37.8)		
Classification of disease				0.000	0.991
IgG+IgA	79	47 (71.2)	32 (71.1)		
Other types	32	19 (28.8)	13 (28.9)		
ISS stage				1.628	0.431
Ⅰ	12	8 (12.1)	4 (8.9)		
II	28	19 (28.8)	9 (20.0)		
Ⅲ	71	39 (59.1)	32 (71.1)		
DS stage				1.873	0.392
Ⅰ	4	3 (4.6)	1 (2.2)		
II	12	9 (13.6)	3 (6.7)		
Ⅲ	95	54 (81.8)	4 (91.1)		
Renal function				10.251	0.001*
Normal	90	60 (91.0)	30 (66.7)		
Abnormal	21	6 (9.0)	15 (33.3)		
Plasma cell ratio				1.631	0.202
<30%	66	36 (54.5)	30 (66.7)		
≥30%	45	30 (45.5)	15 (33.3)		
β2-MG(mg/L)				1.822	0.177
<5.5	63	34 (51.5)	29 (64.4)		
≥5.5	48	32 (48.5)	16 (35.6)		
Hb(g/L)				1.610	0.204
<100	79	44 (66.7)	35 (77.8)		
≥100	32	22 (33.3)	10 (22.2)		
Calcium (mmol/L)				3.917	0.048*
<2.75	89	57 (86.3)	32 (71.1)		
≥2.75	22	9 (13.6)	13 (28.9)		
Albumin				0.535	0.464
<35	57	32 (48.5)	25 (55.6)		
≥35	54	34 (51.5)	20 (44.4)		

*p* < 0.05.

**TABLE 3:** Univariate analysis and multivariate analysis of the response to chemotherapy.

Clinical characteristic	Univariate		Multivariate	
	χ^2^	*p*-value	HR (95% CI)	*p*-value
Age (years)	0.026	0.873		
Gender	0.781	0.377		
Classification of disease	0.055	0.814		
ISS stage	0.200	0.905		
DS stage	3.055	0.217		
Renal function	3.968	0.046*	1.248 (0.401,3.882)	0.702
Plasma cell ratio	0.026	0.873		
β2-MG(mg/L)	0.688	0.407		
Hb(g/L)	1.328	0.249		
ALB	0.192	0.661		
Chemotherapy regimens	3.248	0.072		
NLR	11.197	0.001*	1.430 (0.450,4.539)	0.544
ALI	28.499	0.000*	0.110 (0.035,0.350)	0.000*
PLT	0.481	0.488		
Ca	0.079	0.778		

*p* < 0.05.

### Risk of infection in newly-diagnosed multiple myeloma patients receiving induction therapy

Among the MM patients, 40 (36.04%) presented with infection after induction chemotherapy. The most common site of infection was the respiratory system (32 cases, 80%), followed by the digestive system (3, 7.5%) and the urinary system (2, 5%). In addition, three patients (7.5%) had more than one site of infection. Univariate analysis suggested that ALI and abnormal renal function were significant in predicting infection in NDMM patients (Table 4).

**TABLE 4:** Univariate analysis of infection after chemotherapy in patients with MM.

Risk factors	Group		χ^2^	*p*
	Infected (*n* = 40)	Uninfected (*n* = 71)		
Age (years)			0.003	0.954
<60	24 (60.0)	43 (60.6)		
≥60	16 (40.0)	28 (39.4)		
Gender			0.100	0.752
Male	23 (57.5)	43 (60.6)		
Female	17 (42.5)	28 (39.4)		
Classification of disease			1.221	0.269
IgG+IgA	28 (70.0)	51 (71.8)		
Other types	12 (30.0)	20 (28.2)		
ISS stage			2.529	0.282
Ⅰ	2 (5.0)	10 (14.1)		
II	12 (30.0)	16 (22.5)		
Ⅲ	26 (65.0)	45 (63.4)		
DS stage			3.271	0.195
Ⅰ	0 (0)	4 (5.6)		
II	6 (15.0)	6 (8.4)		
Ⅲ	34 (85.0)	61 (86)		
Renal function			5.006	0.025*
Normal	28 (70)	62 (87.3)		
Abnormal	12 (30)	9 (12.7)		
Plasma cell ratio			0.240	0.624
<30%	25 (62.5)	41 (57.7)		
≥30%	15 (37.5)	30 (42.3)		
β2-MG(mg/L)			1.381	0.240
<3.5	26 (65.0)	38 (53.5)		
≥3.5	14 (35.0)	33 (46.5)		
Hb(g/L)			0.447	0.504
<100	30 (75)	49 (69)		
≥100	10 (25)	22 (31)		
Calcium (mmol/L)				
<2.75	32 (80.0)	57 (80.3)	0.001*	0.097
≥2.75	8 (20.0)	14 (19.7)		
Albumin				
<35	21 (52.5)	36 (50.7)	0.033	0.856
≥35	19 (47.5)	35 (49.3)		
ALI				
<32.7	22 (55.0)	23 (32.4)	5.424	0.020*
≥32.7	18 (45.0)	48 (67.6)		
NLR				
<2.0	16 (40.0)	38 (53.5)	1.872	0.171
≥2.0	24 (60.0)	33 (50.7)		
Chemotherapy regimens			3.795	0.051
Contains bortezomib	36 (90.0)	53 (74.6)		
Does not contain	4 (10.0)	18 (25.3)		
PLT			0.724	0.395
<150	12 (30.0)	27 (38)		
≥150	28 (70.0)	44 (62)		

*p* < 0.05.

## Discussion

The ALI score, comprising BMI, ALB, and NLR, reflects inflammatory immunity and nutritional status. In this study, advanced age, chemotherapy response and serum calcium were independent prognostic factors for OS. Regarding the prognostic potential of ALI in MM patients, a lower ALI score reduced OS, indicating that ALI can serve as an outcome biomarker (Jafri et al., 2013; Park et al., 2017; Mandaliya et al., 2019; Kusunoki et al., 2020; Yin et al., 2021). Similar research has reported that ALI is a predictive marker for PD-L1 inhibitor monotherapy and has stronger predictive ability compared with other analysed parameters, such as NLR and PD-L1 TPS (Mountzios et al., 2021), which have also been confirmed as predictors of OS in NDMM patients treated with bortezomib-based regimens (Romano et al., 2017; Zhou et al., 2018). A retrospective single-centre study in China showed that age, haemoglobin, ALB, serum calcium, β2-MG, LDH, CRP, the ratio of plasma cells and the percentage of abnormal plasma cells in bone marrow were all independent prognostic factors for OS, which is different from our results (Qian et al., 2017). However, the strict inclusion criteria for NDMM patients potentially led to sample selection bias. In addition, the sample size of our study was relatively small, which might also lead to bias in our results. Renal dysfunction is an important characteristic of MM, and the development of renal dysfunction is a negative prognostic factor for MM patients undergoing induction chemotherapy (Li et al., 2021). Our study suggests that low ALI is positively associated with renal dysfunction. Kidney damage often manifests as proteinuria and hypoproteinaemia (Pei et al., 1992), which reduce ALB and result in low ALI. Therefore, ALI also represents a potential negative prognostic factor for patients with MM.

Chemotherapy response is an important prognostic factor in NDMM patients receiving bortezomib-based therapy. Based on univariate analyses, we found that abnormal renal function, high NLR, and lower ALI level are related to poor chemotherapy efficiency. Chen’s research demonstrated that renal function is associated with a high incidence of chemotherapy-related toxicities in metastatic colorectal cancer and even decreases the efficacy of chemotherapy (Chen et al., 2015), which is consistent with our research. In addition, a signature consisting of 11 cytokine genes in the lung environment is able to predict lymph node metastasis and prognosis of lung adenocarcinoma based on IL-8 and TNFα as the top two genes for predicting prognosis (Seike et al., 2007). IL-8 was originally described as a monocyte-derived neutrophil chemotactic factor (MNDCF) that specifically attracts neutrophils (Mukaida et al., 1998), which explains why the NLR value is increased in tumour cells. Hirahara et al. (2019) evaluated the relationship between tumour response and NLR/PLR and found that the score can serve as a new blood predictor of tumour response and prognosis. As discussed above, NLR is related to renal function, and the ALI index is calculated from the NLR value. Therefore, ALI is extremely closely related to the efficacy of chemotherapy. This finding is consistent with our multivariate result that ALI is an independent predictor of chemotherapy effect. Moreover, based on ROC analysis, ALI outperformed other biomarkers of chemotherapy response, including NLR and ALB (Szudy-Szczyrek et al., 2020).

Given that NDMM patients have a high risk of infections at first admission, especially infections with Epstein‒Barr virus (EBV), hepatitis B virus (HBV) and *Escherichia coli* (Lin et al., 2020), we excluded MM patients with concurrent infection from this study. Interestingly, our results highlight that ALI is not only an independent predictive marker of chemotherapy efficacy but also a predictor of susceptibility to infection in NDMM patients during induction chemotherapy. Our study also showed that patients with lower ALI may be more susceptible to infectious complications after receiving induction chemotherapy. Some studies have shown that renal impairment is a risk factor for infections in the early phase of MM or in NDMM patients receiving bortezomib-based induction chemotherapy (Sørrig et al., 2019; Soekojo et al., 2020). We also found that renal failure is a significant risk factor for NDMM patients. Therefore, the presence of low ALI and renal dysfunction may be of greater practical value in predicting infection for MM patients without comorbidities at admission. On the other hand, patients with lower ALI may have a higher NLR value, which may indicate lymphocytopaenia and worse inflammatory immunity and nutritional status (Hirahara et al., 2019), which are significant risk factors for NDMM patients. The incidence of infection after chemotherapy in MM patients is 36.04%, and pulmonary infection is most common, which is consistent with existing research results (O'Brien et al., 2003). Based on data on antimyeloma therapy in previous studies, chemotherapy can increase the risk of bacterial infection, and bortezomib can increase that of influenza infection (Nucci and Anaissie, 2009; Teh et al., 2015). However, we found that chemotherapeutic regimens, including bortezomib, did not increase the risk of infection, which is not consistent with existing research (Nucci and Anaissie, 2009). This finding may be due to our inclusion criteria of MM patients without the presence of comorbidities or multiple organ dysfunctions.

In conclusion, our study confirms that ALI is not only a prognostic biomarker for patients newly diagnosed with MM but also predicts the efficacy of chemotherapy in NDMM patients receiving bortezomib-based therapy. Importantly, those with abnormal renal function have poorer prognosis and are more susceptible to serious infection complications. Therefore, ALI can be adapted in clinical practice to stratify MM patients for future trials. Furthermore, large-scale multicentre prospective studies are required to completely validate our findings.

## Data Availability

The raw data supporting the conclusion of this article will be made available by the authors, without undue reservation.
